# Peace in Guatemala and Immigrant Health in the United States

**DOI:** 10.29024/aogh.2380

**Published:** 2018-11-05

**Authors:** Jeremy C. Green, Eric Adjei Boakye, Amanda Schoening, Michael G. Vaughn

**Affiliations:** 1 Saint Louis University, Department of Health Management and Policy, US; 2 Saint Louis University, Center for Health Outcomes Research, US; 3 Saint Louis University, School of Social Work, US

## Abstract

**Background:**

The civil war between the indigenous Mayans and other Guatemalans lasted for 36 years, killed civilians, decimated villages, and resulted in many refugees. The Guatemalan Peace Agreement of 1996 aimed to alleviate the ongoing conflict. Studies of peace agreements more typically evaluate local political outcomes while neglecting global health outcomes.

**Objective:**

Our research quantified associations between pre-migration exposure to the peace agreement in Guatemala and the post-migration health status of Guatemalan immigrants in the United States.

**Methods:**

We used chi-square tests to compare the distribution of health status before and after peace. We used ordered probit regressions to estimate associations between peace in Guatemala and health in the United States, conditional on the observed distributions of age, age squared, age cubed, and linear time trends before and after peace.

**Findings:**

The study sample included 4,115 female and 5,282 male Guatemalan immigrants between the ages of 15 and 85. The mean age was 38.8 years for females (standard deviation, 14.2) and 35.4 years for males (standard deviation, 12.6). Chi-square tests found statistically significant differences in the distribution of health status before and after the peace agreement, for females (*P* < .001) and males (*P* < .001). In unadjusted results, the peace agreement was associated with a 7.3 percentage point increase in excellent post-migration health for females (95% confidence interval, 4.9 to 9.8) and a 6.0 percentage point increase for males (95% confidence interval, 3.8 to 8.2). In adjusted results, we found that the peace agreement was associated with a 6.1 percentage point increase in excellent post-migration health for females (95% confidence interval, 0.8 to 11.4) and a 5.5-percentage point increase for males (95% confidence interval, 1.0 to 10.0).

**Conclusions:**

The peace agreement in Guatemala was associated with statistically significant improvements in the health status of Guatemalan immigrants to the United States.

Armed conflicts can have negative consequences for many aspects of health and social wellbeing [1,2,3,4], including discrimination [5,6], mental health [7,8,9,10,11], substance abuse [12], hunger [13], health system performance [14], hospital productivity [15], economic development [16], human rights [17], and sexual violence [18]. Central America in general, and Guatemala in particular, is by some measures one of the most violent places in the world [19,20]. The Guatemalan Civil War lasted from 1960 to 1996 [21], killed over 200,000 individuals not including combatants [22], demolished over 400 rural villages between 1981 and 1983 alone [23], and generated over a million refugees [24]. This is one of the longest and most violent conflicts in all of Central America [25]. The Guatemalan Civil War was a continuation of struggles over land and other resources between the native Mayan population and other inhabitants [26]. The population consequences of the Guatemalan Civil War include forced displacements [27] and related migration [28].

In 1996, Guatemala’s national army and other combatants entered into a peace agreement to end violence and alleviate trauma, encourage economic and social development, and discover peaceful pathways to political and social change [29]. The United Nations was involved in Guatemala to a greater degree than they were in other peace agreements and had access to additional funding mechanisms that were not part of regular peacekeeping budgets [30]. The extent of success for the peace agreement is a topic of ongoing debate [31,32], as Guatemala continued to lack an adequate legal system and face continued violence and Mayans continued to live under separate laws. After the peace agreement, Guatemalan society remained divided between a rural indigenous population and a wealthier minority [33]. Despite these limitations, the peace agreement was mediated by the United Nations and viewed as successful by some researchers [34].

Empirical studies of war and peace in Guatemala are occasionally limited by their exclusive focus on the negative effects of war, while neglecting the positive effects of a peace agreement. Previous research by Branas et al. [12: p830] and Puac-Polanco et al. [7: p769] analyzed associations between negative experiences during the civil war and negative outcomes after the civil war. Individuals in these studies reported being attacked with weapons and witnessing serious injuries and deaths during the war and later suffering from depression, anxiety, alcohol-related disorders, and post-traumatic stress disorder. Sabin et al. [11: p639] analyzed data on Guatemalan immigrants in Mexico and found associations of pre-migration exposure to human rights violations and traumatic events related to civil war in Guatemala, with rates of post-migration depression and post-traumatic stress disorder in Mexico.

Another relevant literature seeks to estimate the economic, health, and social consequences of peace agreements [35]. Joshi analyzed variation in comprehensive peace agreements in a sample of 73 post-armed conflict countries between 1989 and 2012 and found that comprehensive peace agreements were associated with declines in child mortality rates [36]. Our research advances the empirical literature on peace agreements by comparing the health status of Guatemalan immigrants who arrived in the United States before and after the Guatemalan peace agreement of 1996. In doing so, our study helps to integrate the literatures on the health effects of armed conflict and on the health effects of peace agreements.

## Methods

Data on Guatemalan immigrants to the United States are derived from the Integrated Public Use Microdata Series, Current Population Survey and Annual Social and Economic Supplement [37]. The Current Population Survey and Annual Social and Economic Supplement data come from a survey of households that is fielded every March by the United States Census Bureau and the United States Bureau of Labor Statistics. Surveys are completed in person and over telephone. These files contain data on Guatemalan immigrants to the United States from 1996 through 2016. While our dataset constitutes a nationally representative sample of the non-institutionalized population of Guatemalan Americans in the United States, it does not contain more detailed information on refugee status and cannot separately identify different types of immigrants beyond classification by country of birth. We analyzed the continuous variable on year of immigration to identify those individuals who were born in Guatemala and arrived in the United States before and after the Guatemalan peace agreement of 1996.

### Study Variables

We compared the health status of Guatemalan immigrants who arrived before and after the peace agreement. For the dependent variable, we analyzed the categorical distribution of health status. The distribution of health status includes five categories – excellent health, very good health, good health, fair health, and poor health. For the key independent variable, we created a variable that was equal to 0 for individuals who were born in Guatemala and immigrated to the United States before the peace agreement, and equal to 1 for individuals who were born in Guatemala and immigrated to the United States after the peace agreement. For covariates in the adjusted specifications, we included age, age squared, age cubed, and linear time trends in year of immigration before and after the peace agreement. In addition to the series of variables included in the peace agreement models, we also analyzed data on years in the United States, to see if health deteriorated over time regardless of the peace agreement.

### Statistical Analysis

The statistical analysis consisted of two steps – a descriptive analysis and a regression analysis. For the descriptive analysis, we tabulated the health status of individuals who were born in Guatemala and subsequently migrated from Guatemala to the United States, for individuals who arrived before the peace agreement and individuals who arrived after the peace agreement separately. We used chi-square tests to describe differences in the distribution of health status between individuals who arrived in the United States before and after the peace agreement in Guatemala.

For the regression analysis, we estimated unadjusted and adjusted associations between the peace agreement and self-rated health status were estimated by fitting ordered probit regression models and then analyzing model predictions [38]. For the unadjusted regression models, we fit ordered probit regressions of the distribution of health status on the peace agreement indicator. For the adjusted regression models, we added a series of covariates to the empirical specifications to adjust for the observed distributions of age, age squared, age cubed, and linear time trends in year of immigration before and after the peace agreement. We estimated incremental effects of arriving in the United States from Guatemala after the peace agreement. In addition to the regression models of the peace agreement and health status, we also fit models of years in the United States, to test the hypothesis that health deteriorated over time, regardless of the peace agreement.

We stratified the data analysis by gender and estimated separate regressions for females and males. Delta method standard errors were computed to estimate 95% confidence intervals [39]. Incremental effects and confidence intervals were estimated using the Current Population Survey supplement probability weights to help generalize the results nationally. We used Stata software, version 14 for data analysis [40]. The Saint Louis University Institutional Review Board reviewed the research, determined that the research was not human subjects research, and exempted the research from further institutional review.

## Results

The study sample included 4,115 females and 5,282 males between the ages of 15 and 85. The mean age was 38.8 years for females (standard deviation, 14.2) and 35.4 years for males (standard deviation, 12.6). We report the results of the data analysis for the study in Tables 1, 2, 3. Table 1 contains the descriptive results and Tables 2 and 3 contain the inferential results.

**Table 1:** Health Status of Guatemalan Immigrants Before and After Peace^a^.

Health	Before Peace, No. (%)	After Peace, No. (%)	*P* Value^b^

**Females (n = 4 115)**			

Excellent	493 (19.42)	373 (23.67)	<.001
Very good	746 (29.38)	537 (34.07)	
Good	903 (35.57)	553 (35.09)	
Fair	305 (12.01)	91 (5.774)	
Poor	92 (3.623)	22 (1.396)	
Total	2 539 (100)	1 576 (100.00)	
**Males (n = 5 282)**			

Excellent	617 (23.34)	721 (27.33)	<.001
Very good	841 (31.81)	969 (36.73)	
Good	899 (34.00)	813 (30.82)	
Fair	208 (7.867)	120 (4.549)	
Poor	79 (2.988)	15 (0.569)	
Total	2 644 (100)	2 638 (100)	

^a^ Table entries are counts and frequencies of individuals born in Guatemala who migrated to the United States before and after the peace agreement. ^b^ *P* values are from chi-square tests.

**Table 2:** Unadjusted Associations Between Peace in Guatemala and Immigrant Health^a^.

Health	Incremental Effect (95% Confidence Interval)	

Gender	Females (n = 4 115)	Males (n = 5 282)

Excellent	0.0732 (0.0487, 0.0978)	0.0596 (0.0376, 0.0817)
Very good	0.0271 (0.0183, 0.0360)	0.0156 (0.00933, 0.0220)
Good	–0.0491 (–0.0660, –0.0323)	–0.0474 (–0.0650, –0.0297)
Fair	–0.0362 (–0.0485, –0.0240)	–0.0203 (–0.0280, –0.0126)
Poor	–0.0150 (–0.0203, –0.00970)	–0.00759 (–0.0109, –0.00426)

^a^ Table entries are incremental effects and 95% confidence intervals from ordered probit regressions of the distribution of health on peace, weighted by the sampling probabilities.

**Table 3:** Adjusted Associations Between Peace in Guatemala and Immigrant Health^a^.

Health	Incremental Effect (95% Confidence Interval)	

Gender	Females (n = 4 115)	Males (n = 5 282)

Excellent	0.0608 (0.00775, 0.114)	0.0546 (0.00954, 0.0996)
Very good	0.0223 (0.00534, 0.0393)	0.0111 (0.00432, 0.0179)
Good	–0.0410 (–0.0772, –0.00483)	–0.0425 (–0.0773, –0.00779)
Fair	–0.0300 (–0.0542, –0.00569)	–0.0170 (–0.0291, –0.00487)
Poor	–0.0122 (–0.0221, –0.00226)	–0.00617 (–0.0103, –0.00202)

^a^ Table entries are incremental effects and 95% confidence intervals from ordered probit regressions of the distribution of health on peace, adjusted for the observed distributions of age, age squared, age cubed, and linear time trends in year of immigration before and after the peace agreement, and weighted by the sampling probabilities.

Table 1 shows the distribution of health status for individuals in the study sample, among those individuals who were born in Guatemala and migrated to the United States before and after the peace agreement, separately. The top panel of Table 1 reports the results for females; the bottom panel of Table 1 reports the results for males. The first column of Table 1 labels the categories of health status on the Likert scale – excellent health, very good health, good health, fair health, and poor health. The second column of Table 1 reports the counts and frequencies of Guatemalan immigrants to the United States by their post-migration health status for the subset of immigrants who arrived before the peace agreement of 1996. The second column of Table 1 reports the counts and frequencies of Guatemalan immigrants who arrived in the United States after the peace agreement of 1996, by their post-migration health status. The final column of Table 1 reports *P* values from chi-square tests of variation in the distribution of post-migration health status between the group of Guatemalan immigrants who arrived in the United States before the peace agreement and the group of Guatemalan immigrants who arrived after the peace agreement.

For both female and male Guatemalan immigrants to the United States, those immigrants who arrived after the peace agreement in Guatemala had an elevated health status, as compared to those immigrants who arrived before the peace agreement in Guatemala. Among females who migrated from Guatemala to the United States, 23.7% of those who migrated after the peace agreement reported excellent post-migration health status, as compared to 19.4% of those who migrated before the peace agreement. Among males, 27.3% of those who migrated after the peace agreement reported excellent post-migration health status, as compared to 23.3% of those who migrated before the peace agreement.

In Table 2, we report the results from a regression analyses of associations between a dichotomous variable for arriving in the United States from Guatemala before and after the peace agreement, and the distribution of post-migration health status. The first column of Table 2 labels the categories of health status, the second column contains the results for females, and the third column contains the results for males. Each cell of the table reports an association between arriving in the United States after the peace agreement in Guatemala, and the distribution of post-migration health status. In the unadjusted results, we found positive associations between the peace agreement in Guatemala and excellent post-migration health status in the population of Guatemalan immigrants in the United States. The peace agreement was associated with a 7.3 percentage point increase in the probability of excellent post-migration health for females (95% confidence interval, 4.9 to 9.8) and a 6.0 percentage point increase in the probability of excellent post-migration health for males (95% confidence interval, 3.8 to 8.2).

In Table 3, we repeat the presentation of regression results the unadjusted analysis in Table 2, and add covariates to the empirical models to adjust the estimated associations between the peace agreement and post-migration health status. After conditioning the regression models on the observed distributions of the covariates – age, age squared, age cubed, and linear time trends in year of immigration before and after the peace agreement – we found positive associations between arriving after the peace agreement and post-migration health status, similar to the results from the unadjusted analysis. In the adjusted models, we found that the probability of excellent post-migration health for females was 6.1 percentage points higher (95% confidence interval, 0.8 to 11.4) for the group of immigrants who arrived after the peace agreement as compared to the group of immigrants who arrived before the peace agreements. Similarly, the probability of excellent post-migration health for males was 5.5 percentage points higher (95% confidence interval, 1.0 to 10.0) for the group of immigrants who arrived after the peace agreement as compared to before the agreement. In Table 4, we report the results of an additional regression analysis, of associations between years in the United States and health status. After adjusting for age, age squared, and age cubed, we found no statistically significant associations between years in the United States and health status.

**Table 4:** Associations Between Years in the United States and Guatemalan Immigrant Health^a^.

Health	Unadjusted Marginal Effect (95% Confidence Interval)	

Gender	Females (n = 4 115)	Males (n = 5 282)

Excellent	–0.00461 [–0.00571, –0.00351]	–0.00437 [–0.00551, –0.00324]
Very good	–0.00181 [–0.00228, –0.00134]	–0.00109 [–0.00143, –0.000756]
Good	0.00304 [0.00232, 0.00375]	0.00345 [0.00255, 0.00436]
Fair	0.00237 [0.00174, 0.00301]	0.00147 [0.00106, 0.00188]
Poor	0.00101 [0.000696, 0.00132]	0.000543 [0.000354, 0.000732]
**Health**	**Adjusted Marginal Effect (95% Confidence Interval)**	

**Gender**	**Females (n = 4 115)**	**Males (n = 5 282)**

Excellent	0.000451 [–0.000858, 0.00176]	–0.000220 [–0.00164, 0.00120]
Very good	0.000174 [–0.000330, 0.000677]	–0.0000537 [–0.000401, 0.000294]
Good	–0.000298 [–0.00117, 0.000570]	0.000174 [–0.000948, 0.00130]
Fair	–0.000230 [–0.000894, 0.000434]	0.0000727 [–0.000398, 0.000543]
Poor	–0.0000971 [–0.000379, 0.000185]	0.0000270 [–0.000148, 0.000202]

^a^ Table entries are average marginal effects and 95% confidence intervals from ordered probit regressions of health status on years in the United States, adjusted for the observed distributions of age, age squared, and age cubed, and weighted by the sampling probabilities.

## Discussion

We compared the self-rated health status of Guatemalan immigrants who arrived in the United States before and after the Guatemalan Peace Agreement of 1996. We found positive associations between arriving in the United States after the peace agreement and the probability of excellent post-migration health status. These results are consistent with positive associations between peace agreements and health outcomes as described in Joshi [36: p6]. A strength of this study is its evaluation of the positive outcomes of peace, which is less common in a literature that usually evaluates the negative outcomes of war. Despite these strengths, our study does have its limitations. The study lacked data on the temporal ordering of migrant locations. We could not distinguish between individuals who were born in Guatemala and stayed in Guatemala until their arrival in the United States from those individuals who were born in Guatemala and lived in one or more other countries before their arrival in the United States. More research is needed to ascertain the complete temporal ordering of migrant locations other than their countries of birth. The study also lacked data on refugee status and identified immigrants solely by country of birth. More research is needed to separately estimate effects of peace agreements on refugee populations as compared to general immigrant populations.
